# BIM-Based Visualization Research in the Construction Industry: A Network Analysis

**DOI:** 10.3390/ijerph16183473

**Published:** 2019-09-18

**Authors:** Zezhou Wu, Changhong Chen, Yuzhu Cai, Chen Lu, Hao Wang, Tao Yu

**Affiliations:** 1 Sino-Australia Joint Research Center in BIM and Smart Construction, Shenzhen University, Shenzhen 518060, China; wuzezhou@szu.edu.cn (Z.W.); 1810332082@email.szu.edu.cn (C.C.); caiyuzhu2016@email.szu.edu.cn (Y.C.); 2 School of Management, Guangzhou University, Guangzhou 510006, China; 3 School of Management Science and Engineering, Central University of Finance and Economics, Beijing 100081, China; hao.wang@cufe.edu.cn; 4 Department of Construction Management, School of Civil Engineering, Harbin Institute of Technology, Harbin 150006, China; yutao2018@hit.edu.cn

**Keywords:** building information modeling (BIM), visualization, scientometric analysis, network analysis

## Abstract

Visualization is one of the main features of Building Information Modeling (BIM). It has many advantages throughout the lifecycle of a construction project, and it has become a timely research topic in recent years. However, no attempt has been made to give a holistic understanding of the existing BIM-based visualization research status. Thus, this study aims to conduct a scientometric analysis of the existing BIM-based visualization literature and to gain a snapshot of the research status during the period 2010–2019. A total of 255 articles were abstracted from the Scopus database. Then, the VOSviewer program was employed to visualize the research status from the perspectives of scholars, countries/regions, journals, articles, and co-occurring keywords. Results revealed that Wang X. published the greatest number of articles, while Cheng J.C.P. received the greatest average normalized citations. Furthermore, *Automation in Construction* was identified as the most influential journal and the article “Building information modeling (BIM): trends, benefits, risks, and challenges for the AEC industry” was the most cited paper. Through the keywords co-occurrence analysis, “virtual reality” and “visual programming language” were identified as the emerging themes in this field. The research findings can provide both researchers and practitioners with a better understanding of the status quo and trends of the BIM-based visualization research.

## 1. Introduction

Building Information Modeling (BIM) is one of the emerging technologies in the construction industry. As defined by the Building Information Model Standard Project Committee, BIM is a digital representation of physical and functional characteristics of a facility [1]. It provides a reliable basis for all decisions in the facility’s lifecycle by sharing information about this facility [2], and it enables different stakeholders to insert, extract, update and modify information to support and reflect their respective intentions [3]. Thus, BIM can keep information accessible and updated in an integrated digital environment, enable stakeholders to fully understand the project information, and improve decision making during project design, construction, management, operation and maintenance [4,5,6]. As the construction industry has been criticized for many reasons, such as for large energy consumption [7,8], resources wastage [9,10], and inefficient communication [11,12], BIM is regarded as a potential solution for the existing problems. Recently, BIM has been employed in many fields, such as in the sustainable design of buildings [13,14,15], lifecycle energy optimization [16,17], e-commerce and e-negotiation [18,19], cost performance improvement [20,21,22], renovation strategy optimization [6,23,24], structural safety diagnosis [25], and productivity improvement [26].

Five main features of BIM were identified by Memon, et al. [27], such as visualization, coordination, simulation, optimization, and plotting. In a real-life project, there are always some places that could not be demonstrated by traditional drawings [28]. However, BIM can make the construction drawings visible to every non-professional to understand the shapes of the project from every aspect [29]. In addition, BIM can create an interactive construction guidance that can be paused at any time and rotated at random angles [30]. When changes occur, they can be compared by simulating different situations. These changes can also be retained as data and become important references for stakeholders to continuously adjust and improve [31]. Zhao [32] and Li, et al. [33] conducted a thorough literature review of global BIM research, and pointed out that the feature of visualization has received a citation burst and become a timely research topic in recent years. However, in current literature, no attempt has been made to give a holistic overview of the existing research status. As Chen, et al. [34] claimed, a systematical examination of the state-of-the-art advancements and emergent trends is imperative to encourage future studies and innovative practices. Thus, in order to have a better understanding of the latest BIM-based visualization research, this study conducts a scientometric analysis of the existing literature which was published during the period 2010–2019.

The following sections of this paper first introduce the research methods used in this study. Then, the networks of the collected literature are analyzed from the perspectives of co-authorship, countries/regions, published journals, citations of articles, and co-occurring keywords. Based on the derived research findings, discussions are presented. Finally, a conclusion section is given at the end of this paper.

## 2. Research Methodology

**Figure 1:**
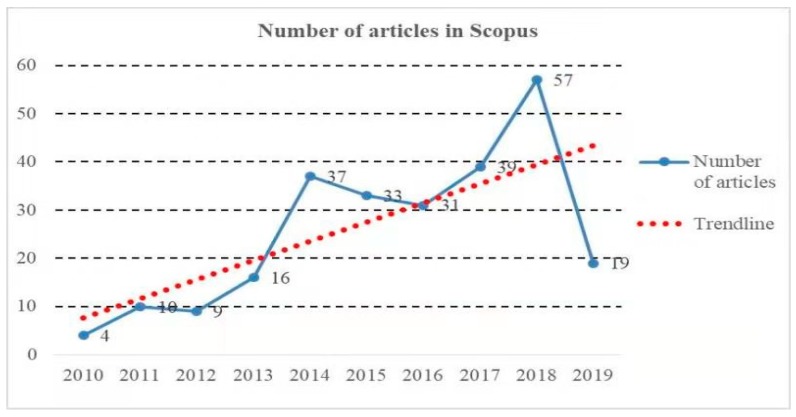
The number of articles in Scopus in 2010–2019.

The BIM-based visualization literature was retrieved from the Scopus database, which consists of substantial important and influential journals in the world. Two rounds of paper retrieval were conducted using the following codes respectively: TITLE-ABS-KEY = (BIM * AND visualize *); TITLE-ABS-KEY = (building information model* AND visualize *). The time range was set from 2010 to 2019, i.e., the past 10 years. Only journal articles were selected for analysis in this study, because journal articles usually provide more comprehensive and high-quality information than the other types of publications [35]. After the two rounds of paper retrieval, a huge number of articles overlapped because BIM and building information modeling were commonly used simultaneously in the same research article. A manual filtering process was then conducted to remove the overlapped papers and irrelevant papers. Finally, a total of 255 bibliographic records were collected. The number distribution of these records is illustrated in Figure 1. It should be noted that, as this study was employed in May 2019, the number of BIM-based visualization studies presented in Figure 1 is not adequate. Overall, the trend of BIM-based visualization research has been increasing since 2010.

After filtering the collected articles, a scientometric analysis was conducted. A scientometric analysis can assist in visualizing the scientific development and structural relationships of a specific research topic [36]. Scientific knowledge maps are usually formulated in the scientometric analysis based on the intersection of applied mathematics, information science and computer science to illustrate the relationships between the development process and structure of knowledge [37,38]. In this study, VOSviewer was employed for formulating the scientific knowledge maps. VOSviewer was selected because it can reduce the controversy caused by subjective judgment and make the research conclusion more scientific [39]. In the VOSviewer, the size of an element depends on the degree of the node, the strength of the connection, etc. The color of an element represents the cluster to which it belongs to, and different clusters are represented by different colors.

In this study, the networks of the retrieved 255 BIM-based visualization journal articles were analyzed from five perspectives, namely, co-authorships, countries/regions, published journals, citation of articles, and co-occurring keywords. The five perspectives were chosen in this study because they are the main bibliography information that the Scopus database can provide, and the networks established from these five perspectives could help researchers to easily grasp the current research status [32]. The evaluated measurements included documents, citations, average citations, average publication year, and average normalized citations. In general, the former three measurements are highly related to each other. The average citations a calculated from dividing the total citations by documents. The average publication year indicates the articles are generally published around a specific year. The average normalized citations quantify the influence—the higher the score, the greater the impact.

## 3. Results and Discussions

As presented in the above section, the networks of the retrieved articles were analyzed from five perspectives, namely, co-authorship, countries/regions, published journals, citation of articles, and co-occurring keywords.

### 3.1. Network of Co-Authorship

**Figure 2:**
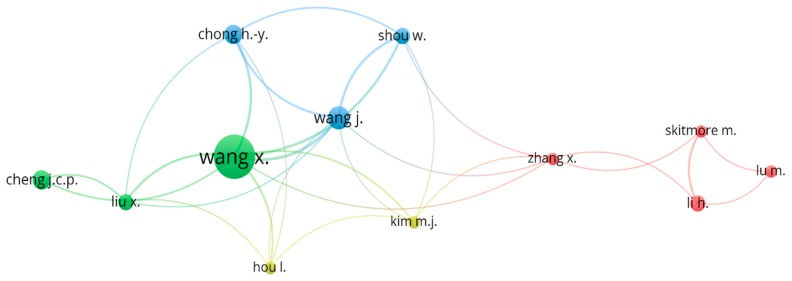
Co-authorship network.

The authorship information of the published articles can be obtained from bibliographic records, and the network of co-authorship can reflect the academic collaborations in the BIM-based visualization field. In this study, the minimum number of articles published and the minimum citations of an author set in the VOSviewer were 3 and 30, respectively. In total, 27 out of 685 authors met the selection criteria. As some of the authors were not connected to each other, a total of 12 authors remained, as presented in Figure 2.

From Figure 2, it can be seen that the identified authors were divided into four groups according to color. In the identified authors, Wang X. has the largest node and has close cooperation with the authors of the other two groups, indicating that Wang X.’s academic influence is the greatest in the field of BIM-based visualization research. The details of the co-authorship network are presented in Table 1.

From Table 1, it can be seen that Wang X. ranked first with 273 citations, followed by Kim M.J. (116), Li H. (103) and Skitmore M. (101). In terms of the average citations, Kim M.J. received the greatest number (39), followed by Skitmore M. (34) and Hou. L. (32). According to the average publication year, an emerging scholar in the field of BIM-based visualization was Cheng J.C.P., whose publications are generally from around 2018. In addition, Cheng J.C.P. had the greatest number of average normalized citations. From Table 1, it is also interesting to find that six of the authors were from Curtin University, revealing that Curtin University played a very important role in BIM-based visualization research.

### 3.2. Network of Countries/Regions

**Figure 3:**
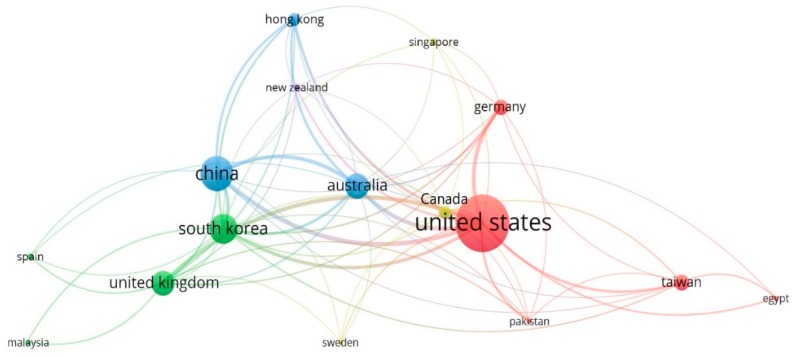
Network of countries/regions.

The contributions of countries/regions were evaluated by VOSviewer with the minimum numbers of documents and citations being set as three and four, respectively. A total of 17 countries/regions met the selection criteria. As one of the items was not connected to the others, a total of 16 countries/regions were presented in Figure 3. From Figure 3, it can be seen that the United States has the largest node, which indicates that the researchers from the United States have made great contributions to the BIM-based visualization research.

The details of the network of countries/regions are presented in Table 2. It is interesting to find that although the Curtin University in Australia played a very important role in the BIM-based visualization research, the overall performance of the United States (70 articles) was better than Australia (27 articles). According to the average normalized citations measurement, it is indicated that Hong Kong received the greatest value, showing that the research outcomes from Hong Kong have had the greatest influence on BIM-based visualization research development.

### 3.3. Network of Published Journals

**Figure 4:**
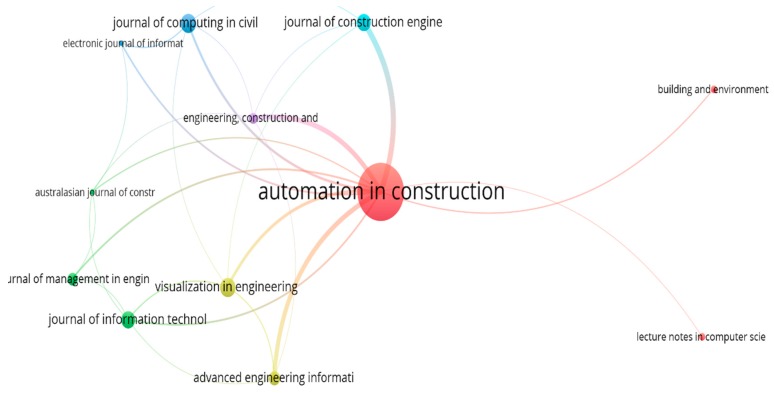
Network of published journals.

The journals that published BIM-based visualization articles were identified and visualized in Figure 4. The minimum number of published papers and the minimum number of citations were set at three and 17, respectively. In total, 14 out of 121 journals met the thresholds. From Figure 4, it can be easily seen that the node of *Automation in Construction* is much larger than the other journals, and it has connections with all the other journals. This indicates that *Automation in Construction* is a leading journal in the field of BIM-based visualization research.

The detailed information of published journals can be obtained from Table 3. From Table 3, it could be verified that *Automation in Construction* is the most influential journal, with the greatest number of documents (40) and the greatest number of citations (1392). In regards to the average number of citations, the *Australasian Journal of Construction Economics and Building* and the *Journal of Construction Engineering and Management* received the greatest number of citations. In terms of the average normalized citations, the *Journal of Management in Engineering*, *Journal of Construction Engineering and Management* and *Automation in Construction* were ranked the top three journals.

### 3.4. Network of Article Citations

**Figure 5:**
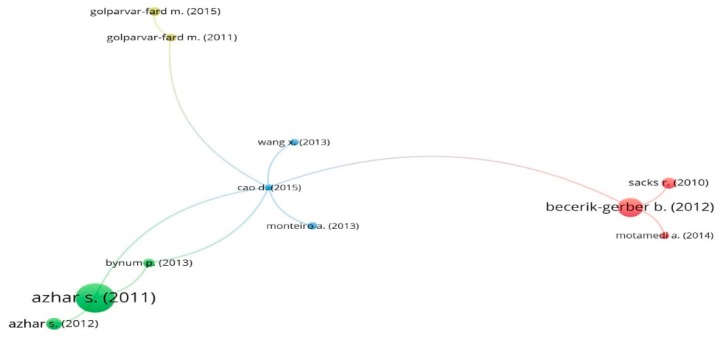
Citation of articles.

The analysis of citations can reflect the most important articles in the BIM-based visualization field. In the VOSviewer, the minimum number of citations was set at 50. Based on the thresholder, a total of 18 out of 255 articles were selected. As some of the items were not connected to each other, the mapping of 11 articles was presented in Figure 5. As can be seen from Figure 5, the most cited article was Azhar [40], receiving the largest node. In addition, though the node of Cao, et al. [41] is not very large, it was located at the center of the map, which means it has the most connections with other highly cited papers.

The details of the article citations information were presented in Table 4. Based on the listed highly cited articles, it can be concluded that, in addition to the general introduction of BIM-based visualization, the existing application included facility management [42,43], lean production management [44], sustainable design [45], progress monitoring [46], augmented reality [47,48], and quantity takeoff [49].

### 3.5. Network of Co-Occurring Keywords

**Figure 6:**
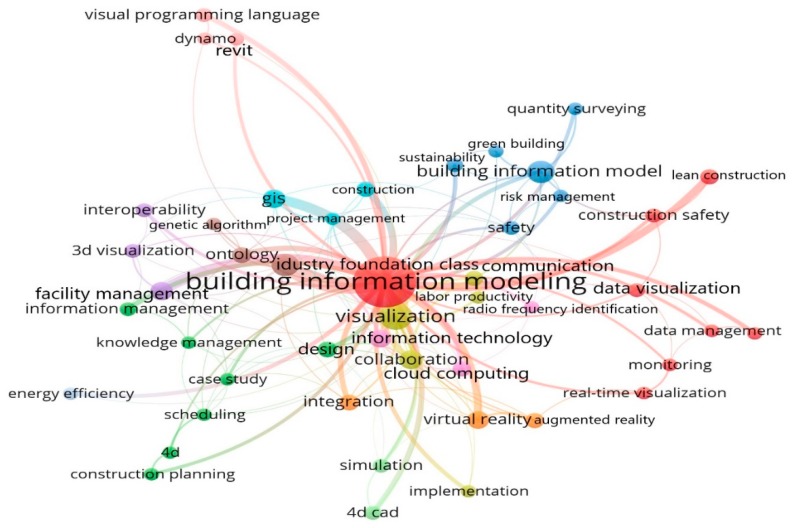
Network of co-occurring keywords.

The frequency of keywords used in the collected articles can reveal the core content of the literature to determine the current research foci and development trends. In this study, the minimum frequency of keywords was set at three. A total of 47 out of 848 keywords met the threshold, and the network of the identified keywords is shown in Figure 6.

From Figure 6, it is not surprising to find that “building information modeling” is the largest node as it is the basis of visualization. The other keywords concerning visualization included “visual programming language”, “real-time visualization”, and “monitoring”, and they were within the same cluster. The other keywords reflected the fields that BIM-based visualization can be applied in, such as “construction safety”, “virtual reality”, “scheduling”, “energy efficiency”, “knowledge management” and “information management”.

The details of keyword frequency are shown in Table 5. The number of occurrences echoed with the illustrations from Figure 6. According to the average citations, the following keywords including “information technology”, “collaboration”, “lean construction”, “sustainability”, and “interoperability” received more attention. The average year of publication indicated the year in which these keywords are concentrated. For example, papers related to “virtual reality” and “visual programming language” were published around 2017, indicating that these emerging themes received researchers’ attentions in recent years and might represent future research directions. The average normalized citation values indicated that these keywords have gained extensive attention, especially “information technology” which has the greatest number of average citations.

## 4. Discussions

Based on Figure 6 and Table 5, BIM-based visualization technology can be combined with many research domains. From a holistic perspective, three main research domains were identified, namely, safety and health, project management, and sustainable development.

The BIM-based visualization can enhance safety and health in construction projects. In recent years, occupational health and safety has been recognized as a primary concern in the construction industry [51,52,53,54]. With the aid of BIM software, second development is available to integrate with external sensors, which can provide a holistic and real-time monitoring of the safety and health environment of construction sites. For example, Cheung [55] developed a real-time construction safety monitoring system for hazardous gas by integrating BIM and a wireless sensor network. Dong, et al. [56] integrated real-time location systems and sensors with BIM to avoid the misuse of personal protective equipment so as to reduce the occurrence of critical accidents and diseases. In subway spaces, Marzouk and Abdelaty [57] visualized the readings of air temperature and humidity levels based on BIM and a wireless sensor network to monitor thermal comfort and to predict problems in the subway heating, ventilation and air conditioning (HVAC) systems. In terms of noise, Wei, et al. [58] predicted noise spatial distribution by collecting scattered data from wearable noise sensors in combination with BIM, aiming to prevent construction workers from noise-induced hearing loss. In addition to the real-time monitoring of the safety environment, BIM can also integrate with virtual reality to enhance construction safety. Virtual reality has a critical feature of immersion, and thus there is a sense of stereoscopic depth. At present, it is not uncommon to construct a virtual reality experience room to simulate on-site construction and to avoid the potential safety problems [59].

The project management can be facilitated by BIM-based visualization as well. Completing a project in due time is an important target of project management. Ivson [60] developed a CasCADe visualization system, which could make task sequencing and spatio-temporal simultaneity immediately apparent, to review real-world construction plans. Material preparation is essential for building construction. Singh [61] utilized BIM data as input to generate the visualization of the formwork quantity and schedule, which could reduce the efforts expended on formwork design. During the lifecycle of a construction project, efficient collaborations among stakeholders are essential for the success of a project. However, different stakeholders may be not familiar with professional knowledge in other areas. Wang, et al. [62] established a collaborative working platform which can help to visualize different stakeholders’ opinions and conducted a case study to verify the effectiveness of the platform.

BIM-based visualization can also be used to improve the sustainability of a project. Jrade [63] integrated BIM with LCA tools for designing sustainable building projects. By using the novel methodology, the environmental impacts can be evaluated and visualized at the conceptual stage. Kensek [64] further developed a software-assisted approach to characterize whether a proposed building design would align with the Leadership in Energy and Environmental Design (LEED) criteria. Olawumi [65] revealed that the visualization feature of BIM can ensure real-time sustainable design and multi-design alternatives, which could facilitate sustainable materials and components selection, and reduce material wastage. Bonenberg [66] stated that BIM can visualize digital building models so as to improve air flow analysis and building sunshine ecosystems.

## 5. Conclusions

Visualization is a main feature of BIM, and it has been recognized as a timely research topic in recent years. In order to give a holistic overview of the existing research status, this study utilized VOSviewer to map the networks of the relevant articles published between 2010 and 2019 from five perspectives, namely, co-authorship, countries/regions, journals, article citations, and co-occurrence keywords. The results showed that Wang X. published the greatest number of articles while Cheng J.C.P. received the greatest average normalized citations. In terms of the countries/regions, the United States has made great contributions to BIM-based visualization development. *Automation in Construction* was identified as the most influential journal in the field of BIM-based visualization. The article “Building information modeling (BIM): trends, benefits, risks, and challenges for the AEC industry” received the most citations in the research field. The analysis of keyword co-occurrence showed that “virtual reality” and “visual programming language” were the emerging themes that received researchers’ attentions in recent years. Based on the identified most used keywords, three main research domains of BIM-based visualization were identified, namely safety and health, project management, and sustainable development.

The research findings of this study could enable an overall picture of the current status of BIM-based visualization research for both researchers and practitioners. However, this study has limitations. For example, only English articles were analyzed in this study, but there might be excellent studies in other languages. Thus, if there is a necessity to investigate the BIM-based visualization research status in a specific country/region, future research can be conducted using similar methods.

## Figures and Tables

**Table 1:** Details of co-authorships.

Scholar	Affiliation	Documents	Citations	Avg. Citations	Avg. Pub. Year	Avg. Norm. Citations
Wang X.	Curtin University	13	273	21	2015	1.18
Wang J.	Curtin University	6	81	14	2015	0.71
Cheng J.C.P.	Hong Kong University of Science and Technology	5	40	8	2018	2.05
Chong H.Y.	Curtin University	5	46	9	2015	0.92
Li H.	Hong Kong Polytechnic University	4	103	26	2016	1.51
Liu X.	Curtin University	4	38	10	2016	1.04
Shou W.	Curtin University	4	78	20	2014	1.02
Hou L.	Curtin University	3	96	32	2014	0.92
Kim M.J.	Kyung Hee University	3	116	39	2014	1.22
Lu M.	Hong Kong Polytechnic University	3	48	16	2016	1.28
Skitmore M.	Queensland University of Technology	3	101	34	2016	1.71
Zhang X.	City University of Hong Kong	3	49	16	2016	0.97

**Table 2:** Details of countries/regions.

Country/Region	Documents	Citations	Avg. Pub. Year	Avg. Citations	Avg. Norm. Citations
United States	70	1760	2015	25	1.20
China	39	256	2017	7	0.79
South Korea	33	687	2016	21	1.06
Australia	27	743	2015	28	1.42
United Kingdom	26	439	2016	17	1.09
Taiwan	16	117	2016	7	0.71
Germany	15	235	2017	16	1.03
Canada	12	160	2017	13	0.93
Hong Kong	12	168	2017	14	1.98
New Zealand	6	62	2017	10	0.81
Singapore	6	14	2018	2	1.07
Spain	5	57	2016	11	0.31
Egypt	4	76	2015	19	0.87
Malaysia	4	10	2017	3	0.23
Pakistan	4	79	2015	20	1.20
Sweden	4	59	2015	15	0.74

**Table 3:** Details of published journals.

Full Name of Journal Sources	Documents	Citations	Avg. Citations	Avg. Norm. Citations
*Automation in Construction*	40	1392	35	1.90
*Journal of Computing in Civil Engineering*	11	222	20	0.98
*Visualization in Engineering*	11	73	7	0.45
*Journal of Construction Engineering and Management*	10	551	55	1.93
*Electronic Journal of Information Technology in Construction*	10	121	12	0.79
*Advanced Engineering Informatics*	8	70	9	0.89
*Journal of Management in Engineering*	7	136	19	2.08
*Engineering, Construction and Architectural Management*	6	17	3	0.34
*Building and Environment*	4	56	14	1.13
*Lecture Notes in Computer Science* (including subseries lecture notes in artificial intelligence and lecture notes in bioinformatics)	4	18	5	0.27
*Australasian Journal of Construction Economics and Building*	3	196	65	1.52
*Electronic Journal of Information Technology in Construction*	3	125	42	0.74

**Table 4:** Details of articles citations.

Article	Title	Citations	Norm. Citations
Azhar [40]	Building information modeling (BIM): trends, benefits, risks, and challenges for the AEC industry	465	6.0944
Becerik-Gerber, Jazizadeh, Li and Calis [42]	Application areas and data requirements for BIM-enabled facilities management	266	4.1926
Azhar, et al. [50]	Building information modeling (BIM): now and beyond	147	2.317
Sacks, Radosavljevic and Barak [44]	Requirements for building information modeling based lean production management systems for construction	132	2.9333
Bynum, Issa and Olbina [45]	Building information modeling in support of sustainable design and construction	98	2.2529
Golparvar-Fard, Pena-Mora and Savarese [46]	Automated progress monitoring using unordered daily construction photographs and IFC-based building information models	95	4.1633
Golparvar-Fard, Peña-Mora and Savarese [47]	Integrated sequential as-built and as-planned representation with D^4^ AR tools in support of decision-making tasks in the AEC/FM industry	86	1.1271
Monteiro and Poças Martins [49]	A survey on modeling guidelines for quantity takeoff-oriented BIM-based design	75	1.7241
Wang, Love, Kim, Park, Sing and Hou [48]	A conceptual framework for integrating building information modeling with augmented reality	72	1.6552
Motamedi, Hammad and Asen [43]	Knowledge-assisted BIM-based visual analytics for failure root cause detection in facilities management	67	4.044
Cao, Wang, Li, Skitmore, Huang and Zhang [41]	Practices and effectiveness of building information modelling in construction projects in china	63	2.761

**Table 5:** Details of co-occurring keywords.

Keywords	Occurrences	Avg. Pub. Year	Avg. Citations	Avg. Norm. Citations
Building Information Modeling	187	2016	19	1.09
Visualization	28	2015	11	0.67
Building Information Model	17	2016	22	0.69
Industry Foundation Class	17	2016	13	1.07
Collaboration	10	2014	67	1.17
Facility Management	9	2016	11	2.21
GIS	9	2014	30	1.41
Information Technology	8	2015	87	2.22
Virtual Reality	8	2017	14	0.88
Design	6	2016	21	0.65
Integration	6	2014	12	0.63
Ontology	6	2017	21	1.31
Augmented Reality	5	2015	35	1.06
Cloud Computing	5	2015	19	1.90
Communication	5	2014	8	0.48
Construction	5	2016	23	0.85
Lean Construction	5	2015	42	2.00
3D Visualization	4	2016	16	0.58
4D CAD	4	2014	27	1.42
Construction Safety	4	2016	23	1.21
Data Visualization	4	2016	10	0.66
Information Management	4	2015	26	2.01
Interoperability	4	2014	39	0.99
Safety	4	2016	10	0.71
Simulation	4	2016	10	0.52
Sustainability	4	2015	40	1.64
Visual Programming Language	4	2017	4	0.36
